# Acute red eye and back pain as a presentation for systemic illness: case report

**DOI:** 10.1186/1471-2415-6-31

**Published:** 2006-09-22

**Authors:** Jonathan M Smith, Philip G Griffiths, Scott G Fraser

**Affiliations:** 1 Ophthalmology, Sunderland Eye Infirmary, Sunderland, UK; 2 Ophthalmology, Royal Victoria Infirmary, Newcastle upon Tyne, UK

## Abstract

**Background:**

Acute red eye is a common presentation in both primary and secondary care. Presentation in combination with other systemic symptoms can indicate serious underlying pathology.

**Case presentation:**

73-year-old lady presenting with endogenous endophthalmitis and thoracic discitis secondary to sub-acute bacterial endocarditis.

**Conclusion:**

Acute red eye in combination with systemic symptoms requires immediate investigation. If endogenous endophthalmitis is diagnosed, a source of sepsis should be comprehensively investigated and referral made to individual specialities if necessary.

## Background

Acute painful red eye is a common presenting complaint. The differential diagnosis is broad and consideration needs to be given to both local and underlying systemic conditions [1].

We report the presenting combination of an acute red eye and new onset back pain, in a well 73-year old lady diagnosed with endogenous endophthalmitis and thoracic discitis secondary to a subacute bacterial endocarditis. This case emphasises the need for consideration of systemic illness as a cause for red eye, especially in combination with other symptoms.

## Case presentation

A 73-year old lady was referred to the Ophthalmic Accident and Emergency Department with a five-day history of decreased visual acquity and floaters in the vision of her left eye. The patient had no previous medical or ophthalmic history and had been well over the preceding weeks, other than a treated urinary tract infection. Initial examination revealed visual acuity in the right eye – 6/9, in the left – finger counting. Conjunctival chemosis was present in the left eye, posterior synechiae and a cloudy vitreous with no fundal view.

Ocular ultrasound and vitreal biopsy (no growth) enabled a diagnosis of endogenous endophthalmitis to be made. Treatment with intravitreal and systemic antibiotics improved the patient's symptoms and she was discharged. Investigation into the source of the sepsis before discharge included an ultrasound of her abdomen, which revealed no intra-abdominal focus.

Two days later the patient was readmitted as a medical emergency with symptoms of acute left ventricular failure and worsening lower back pain. Examination demonstrated marked tenderness in the lower thoracic region, previously undocumented systolic and diastolic murmurs and signs of pulmonary oedema. Investigation revealed elevated inflammatory markers, blood cultures grew Group B *Streptococcus *and MRI showed a discitis at T11/12 consistent with infection.

**Figure 1:**
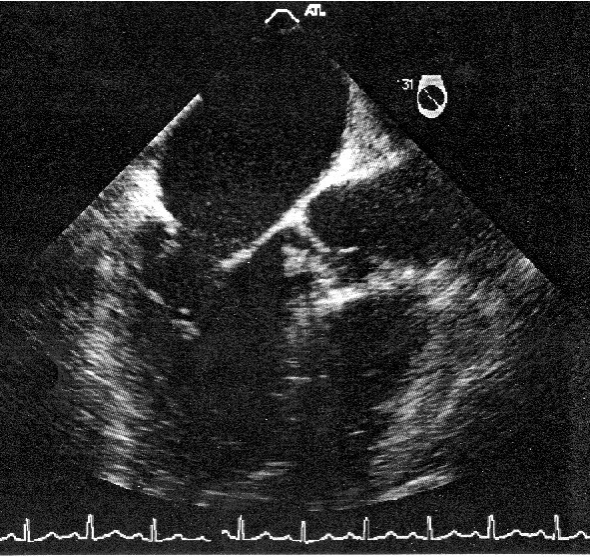
Transoesophageal echocardiography image showing a vegetation on the right coronary cusp of the aortic valve prolapsing during diastole.

Transoesophageal Echocardiography showed a mobile echo dense mass on the right coronary cusp of the aortic valve (See Figure 1).

After a six-week course of intravenous antibiotics and two months of oral antibiotics a successful aortic valve replacement was performed. The discitis resolved on the above regimen with no sequelae and vision in the left eye improved to 6/9.

## Discussion and conclusion

Endogenous endophthalmitis accounts for a small proportion of all cases of endophthalmitis [2]. Review of the literature has shown that certain underlying medical conditions are associated with endogenous endophthalmitis, the most common of which is endocarditis [3]. Others include kidney/urinary tract infection, gastrointestinal abscess, cellulitis, meningitis, liver abscess [3] and septic arthritis [4]. Causative organisms are also widespread, including both gram negative [5] and positive. Group B *Streptococcus *accounts for approximately 7% of cases of endogenous endophthalmitis [3]. Prompt identification of the causative organism and an active therapeutic approach, including intravitreal antibiotics and vitreoretinal surgery, can preserve the sight in the infected eye [6].

The combination of endophthalmitis and discitis in the presentation of endocarditis is unusual, and to our knowledge has not previously documented in the literature. The case demonstrates important points for both general physicians and ophthalmologists:

• The presentation of an acute red eye with systemic symptoms, particularly new onset back pain, can indicate a serious underlying medical condition.

• If systemic illness is suspected in combination with acute red eye, referral to ophthalmology should be sought.

• If endogenous endophthalmitis is diagnosed then a source of sepsis should be investigated as comprehensively as possible. Dependent on systemic findings this may require referral to individual specialities. In this case further investigation into the underlying cause of the endophthalmitis during the initial admission could have led to earlier diagnosis of the underlying endocarditis, and perhaps reduced subsequent sequelae.

## Competing interests

All authors of this article declare they have no conflict of interest.

## Authors' contributions

All authors were involved in patient management or writing of the manuscript.

## Pre-publication history

The pre-publication history for this paper can be accessed here:


